# The Relationship between Higher Chronic Opioid Therapy Dose and Specific Personality Traits in Individuals with Chronic Pain

**DOI:** 10.1155/2021/9946067

**Published:** 2021-06-28

**Authors:** Amanda McIntyre, Swati Mehta, Danielle Vanderlaan, Keith Sequeira, Eldon Loh, Robert Teasell

**Affiliations:** ^1^Parkwood Institute Research, Parkwood Institute, London, ON, Canada; ^2^Department of Physical Medicine and Rehabilitation, Schulich School of Medicine and Dentistry, University of Western Ontario, London, ON, Canada; ^3^Physical Medicine and Rehabilitation, St. Joseph's Health Care, Parkwood Institute, London, ON, Canada

## Abstract

**Objective:**

To evaluate the relationship between opioid use and specific personality traits among individuals with chronic pain stratified by morphine equivalent doses (MEQ).

**Design:**

Observational cohort study. *Setting*. Chronic pain outpatient clinic in Canada (2017–2019). *Patients*. Participants were included if they (1) were at least 18 years old, (2) had been diagnosed with chronic pain (pain >3 months), and (3) were able to read and write in English. *Interventions*. None. *Main Outcome Measures*. Completion of the following outcome measures: Acceptance and Action Questionnaire, Anxiety Sensitivity Index, Brief-Coping with Problems Experience 28-item, Brief Pain Inventory Short Form, CAGE-AID substance misuse screening tool, EuroQol-5D, Generalized Anxiety Disorder 7-item, and Patient Health Questionnaire 9-item. One-way analysis of variance compared outcomes between MEQ groups.

**Results:**

215 individuals (64.2% female) were included with a mean age of 52.7 ± 11.7 years and time since pain onset of 14.1 ± 10.2 years (range 1–45). There were no significant differences between MEQ groups with respect to sociodemographic and clinical health variables except for gender and employment status and time since pain onset. After controlling for gender, time since pain onset, and average pain severity, patients with MEQ 90+ mg had significantly higher scores for experiential avoidance and anxiety sensitivity in addition to increased pain interference, greater depressive and anxiety symptoms, more dysfunctional coping, and poorer QoL than those with MEQ 1–89 mg or MEQ 0 mg.

**Conclusions:**

Compared to individuals using no or lower-dose opioids to treat chronic pain, those using high-dose opioids had higher scores on two maladaptive personality traits (i.e., anxiety sensitivity and experiential avoidance) which was associated with poorer mood, greater pain interference, lower quality of life, and dysfunctional coping. These maladaptive personality traits may help to explain how individuals with chronic pain utilize higher doses of opioid analgesics.

## 1. Introduction

Chronic pain is a debilitating condition that affects approximately 19% of the Canadian population [1]. Chronic pain is defined as pain that lasts longer than three months [2] and is often exacerbated by environmental, sociodemographic, and individual factors. As such, it can have a significant effect on a person's mood, activities of daily living, and quality of life [3].

Previous research has explored the role of obsessive personality traits on coping ability among individuals with chronic pain [4, 5]. The findings showed that there exists a high-risk group whose level of obsessive traits significantly correlated with impaired mood and coping [4]. These trait-like factors, which are stable personality features, have been shown to have a negative influence on outcomes such as mood and disability among those with chronic pain. Experiential avoidance (EA) and anxiety sensitivity (AS) are two traits shown to negatively impact these outcomes. EA has been broadly defined as attempts to avoid thoughts, feelings, emotions, memories, and other internal sensations even when doing so creates harm in the long-term, whereas AS is the fear of anxiety-related sensations among individuals with chronic pain. While personality traits have historically been considered stable, studies over the last few decades have demonstrated that they can change over time and development, or with exposure to situation stress [6, 7]; as such, the severity of maladaptive personality traits can predict changes in health, disability, and quality of life. For example, EA and AS have been shown as important in predicting disability among patients with chronic pain [8].

Recent work by Mehta et al. [4] demonstrated that the interaction of AS and EA significantly impacted pain-related disability over a one-year time frame in patients referred to a chronic pain specialist and that individuals with high levels of EA and AS have a more unfavorable disability outcome over time. It is suggested that these individuals struggle with trying to maintain premorbid levels of activities; failure to “keep up” results in greater stress and depressive symptoms and poorer coping. It is anticipated that greater pain interference and higher stress levels associated with pain-related limitations cause individuals to more aggressively seek out treatment options so that they can reestablish premorbid levels of activity and productivity.

Treatment for various chronic pain conditions often utilizes a broad spectrum of nonpharmacologic and pharmacologic options, including opioid analgesics [9]. Despite management with recommended nonpharmacologic and pharmacologic treatments, many individuals with chronic pain struggle to reestablish their premorbid level of activity and productivity. Individuals in these circumstances may experience high levels of stress and dysphoria when faced with pain-related limitations and may more aggressively pursue treatment for unrelenting chronic pain including opioids. The 2017 Guideline on Opioid Therapy for Chronic Noncancer Pain [10] suggests that physicians limit opioid analgesic dosing when beginning opioid therapy to no more than 90 mg morphine equivalent dose. Over the last several decades, the use of opioid analgesics including higher doses for managing chronic pain became popular and widespread due to the drug's inherent potency, increased flexibility in prescribing guidelines for physicians, and aggressive pharmaceutical marketing. However, as their popularity increased, there was an associated increase in opioid misuse [11]. Opioid prescription and use remain controversial due to its side effect profile and limited associated functional gains [12, 13]. There is a risk for misuse, physical dependence, and addiction, and prescription opioids have been an important factor in the opiate epidemic.

Numerous studies have assessed the relationship between psychological variables and general chronic pain [14–17] or opioid misuse [18–22]. There are few studies that have specifically assessed how personality traits and psychological variables differ between individuals using different doses of opioids in a chronic pain population. Individuals on high-dose opioids will rarely start off at a high dose; most individuals using opioids for chronic pain remain on largely smaller doses [23, 24]. Even when pain persists, they often do not progress to higher doses. We were interested in whether the same personality factors (AS and EA) that significantly influence chronic pain disability and mood are also related to opioid analgesic dosage. Therefore, the purpose of this exploratory study was to evaluate the relationship between opioid dosage and these same problematic personality traits as well as psychosocial variables among individuals with chronic pain stratified by morphine equivalent doses (i.e., 0 mg, 1–89 mg, and 90+ mg MEQ).

## 2. Materials and Methods

This study used a prospective, observational cohort design with a chronic pain population. Ethics was approved by the Ethics Review Board at Western University prior to study initiation.

### 2.1. Recruitment

Participants were recruited from a chronic pain outpatient clinic in Ontario, Canada, between May 2017 and September 2019; no restriction was placed on sample size. Patients were recruited for the study during an appointment with their physiatrist. Candidates were provided a letter of information from their physiatrist. After the appointment, if the patient expressed interest in the study, they were directed to a research assistant stationed in the outpatient clinic. If the patient agreed to participate, they consented to the study. Participants were only included if they (1) were at least 18 years old, (2) had been diagnosed with chronic pain (pain >3 months), and (3) were able to read and write in English. Participants were excluded if they had a cognitive impairment and a psychiatric disorder or were unable to provide informed consent, as determined by their physiatrist.

### 2.2. Data Collection

The following sociodemographic variables were collected by the research assistant: age, gender, employment status, level of education, living arrangement, and race/ethnicity. The research assistant provided the participant with a package of questionnaires. Within the package were the following outcome measures: Acceptance and Action Questionnaire (AAQ)^18^, Anxiety Sensitivity Index (ASI)^19^; Brief-Coping with Problems Experience 28 item (Brief COPE-28)^20^, Brief Pain Inventory Short Form (BPI-SF)^21^, CAGE-AID substance misuse screening tool^22^, EuroQol-5D (EQ5D)^23^, Generalized Anxiety Disorder 7 item (GAD-7)^24^, and Patient Health Questionnaire 9 item (PHQ-9^25^). After the participant completed the questionnaires, the research assistant obtained the following clinical variables from the patient's chart: time since pain onset, length of clinic enrolment, type of pain (i.e., nociceptive, neuropathic, and mixed), type of opioids used, use of nonopioid medications, use of cannabis, and morphine equivalent (MEQ) dose.

### 2.3. Data Analysis

All data were organized and analyzed in SPSS version 23.0 (Chicago, IL); significance was set at *p* < 0.05. Counts (percentage) and/or means with standard deviations were reported for all variables and outcome measures. Participants were categorized into three groups according to their MEQ dose: 0 mg, 1–89 mg, and 90+ mg. One-way analysis of variance and Bonferroni-corrected post hoc *t*-tests were used to compare continuous variables between MEQ groups, controlling for significant sociodemographic and clinical health variables. Pearson's chi-squared analysis was used to compare categorical variables between MEQ groups. Adjusted residuals (z scores) for each pairwise comparison were converted to a chi-squared value and then *p* values. These pairwise (converted) *p* values were compared against the Bonferroni-corrected *p* value to determine significance. The corrected *p* value was determined by dividing the overall significance at *p* = 0.05 by the number of pairwise comparisons.

## 3. Results

### 3.1. Sociodemographic Information

A total of 220 individuals were approached, and all but 2 consented to participate in the study; however, 3 individuals had incomplete data, so 215 individuals were included for analysis. A descriptive analysis of the sociodemographic variables for the overall sample and for each MEQ group is provided in Table 1. There were no significant differences between MEQ groups with respect to sociodemographic variables except for gender and employment status. There were more females than males in the MEQ 0 mg group (*p* = 0.005) and more males than females in the MEQ 90+ mg group (*p* < 0.001); there were approximately equal numbers of males and females in the MEQ 1–89 mg group. The MEQ 90+ mg group had a greater number of individuals who were unemployed than the other groups (*p* < 0.001).

### 3.2. Clinical Health Information

Clinical health variables for the entire sample and for each MEQ group are shown in Table 2. The classification of injuries sustained was highly variable; for nearly half of all patients, chronic pain resulted from either a motor vehicle accident (34.9%) or a workplace injury or accident (12.6%). For all remaining patients, injury classifications were diverse: neurological conditions, physical assault, slip and fall events, sports injuries, osteoarthritis, disc degeneration, and concussion. For 6.9% of patients, the classification was unspecified back/neck pain and 6.5% were missing. All patients experienced pain for at least one year prior to entering the study. There were no significant differences between MEQ groups with respect to the type of pain, use of nonopioids, and use of cannabis. However, time since pain onset was significantly different between groups, positively and linearly related to MEQ dose (*p* < 0.001). The same association was found with a length of clinic enrolment whereby individuals with greater MEQ doses had been enrolled in the clinic for a longer length of time (*p* < 0.001). There were significant differences between the MEQ 1–89 mg and MEQ 90+ mg groups with respect to the type of opioid used for pain management. Patients in the high-dose MEQ group (90+ mg) used significantly more oxycodone/extended-release oxycodone (*p* < 0.001), morphine/extended-release morphine (*p* = 0.039), and fentanyl (*p* = 0.007) than those in the MEQ 1–89 mg group. Conversely, those in the low-dose MEQ group (1–89 mg) used significantly more tramadol/tramadol with acetaminophen (*p* < 0.001) and codeine/codeine with acetaminophen (*p* = 0.002) than those in the MEQ 90+ mg group.

### 3.3. Outcome Measure Data

**Figure 1:**
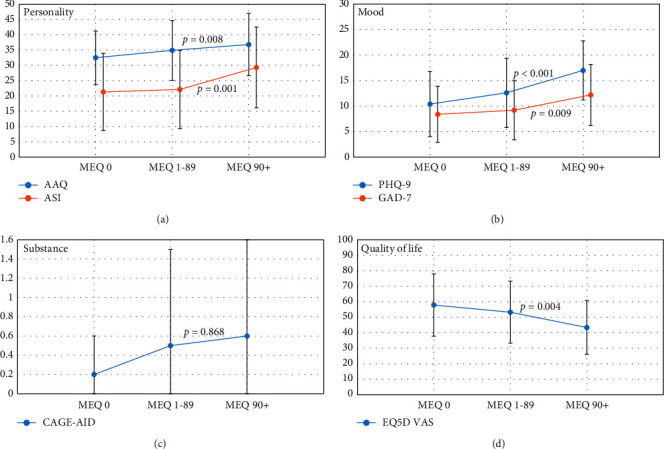
Mean and standard deviation for outcome measure scores among the three MEQ groups (0, 1–89, 90+) for (a) personality traits (AAQ, ASI), (b) mood (PHQ-9, GAD-7), (c) substance misuse (CAGE-AID), and (d) quality of life (EQ5D).

All outcome measure data and comparisons between groups (controlled for gender, time since pain onset, and average pain rating using item 5 on the BPI-SF) are shown in Table 3 and depicted in Figure 1.

*Pain Interference*. After controlling for gender, time since pain onset, and average pain rating (item 5 on the BPI-SF), pain interference (items 9a-g) was significantly different between groups; individuals in the MEQ 90+ mg group had significantly greater pain interference in comparison to individuals in the MEQ 1–89 mg and MEQ 0 mg groups (*p* < 0.05 for both).

*Personality Traits*. After controlling for gender, time since pain onset, and average pain rating, mean scores on both AAQ and ASI were shown to significantly differ between MEQ groups (*p* = 0.001 and *p* = 0.008, respectively). Patients in the high-dose group (MEQ 90+ mg) had significantly greater levels of experiential avoidance (AAQ) as compared to the MEQ 0 mg group (*p* < 0.05). Similarly, those in the MEQ 90+ mg group had greater levels of anxiety sensitivity (ASI) compared to the MEQ 1–89 mg and MEQ 0 mg group (*p* < 0.05). Chi-squared analysis showed that there were significantly fewer individuals with mild anxiety sensitivity in the MEQ 90+ mg group compared to the other groups (*p* = 0.004).

*Mood*. After controlling for gender, time since pain onset, and average pain rating, mean scores on the PHQ-9 and GAD-7 were shown to be significantly different between groups (*p* < 0.001 and *p* = 0.009, respectively). Individuals in the high-dose MEQ group (90+ mg) had higher levels of depressive and anxiety symptoms than those in the MEQ 1–89 mg and MEQ 0 mg groups (*p* < 0.05 for all). Further, we examined the proportion of individuals in each depressive symptom severity category across the MEQ groups. Individuals in the MEQ 90+ mg group had significantly fewer cases of nonminimal depressive symptoms (*p* = 0.003) and significantly more cases of moderately severe (*p* = 0.002) and severe (*p* = 0.001) depressive symptoms.

*Substance Misuse, QoL, and Coping*. After controlling for gender, time since pain onset, and average pain rating, there were no significant differences between MEQ groups with respect to mean CAGE-AID scores (*p* = 0.868) or the proportion of individuals identified to be at risk or not at risk for substance misuse (*p* = 0.790). After controlling for gender, time since pain onset, and average pain rating, QoL was shown to be significantly inversely related to MEQ dose (*p* = 0.004) with individuals in the MEQ 90+ mg group having poorer QoL than those in the MEQ 1–89 mg or MEQ 0 mg groups (*p* < 0.05 for both). After controlling for gender, time since pain onset, and average pain rating, mean scores for problem-based coping and emotion-focused coping were not significantly different between MEQ groups; however, those in the MEQ 90+ mg group had significantly greater dysfunctional coping scores than those in the MEQ 0 mg group (*p* = 0.008).

## 4. Discussion

Our results show that even after controlling for gender, time since pain onset, and average pain severity, patients taking high doses of opioids (MEQ 90+ mg) had significantly greater EA and AS than those taking opioids within the recommended guidelines (MEQ 1–89 mg) or not at all (MEQ 0 mg). Additionally, those taking the highest opioid doses had greater depressive symptoms, anxiety symptoms, and dysfunctional coping ability with subsequently poorer QoL. Interestingly, there were no differences in substance misuse scores on the CAGE-aid, so substance misuse was likely not the primary driver of higher opioid doses.

We were most interested in examining specific personality or dispositional traits and their association with opioid dosing among patients with chronic pain. Dispositional traits are generally considered to be fixed and present premorbidly [25]; in our case, this suggests that traits are stable prior to and after chronic pain onset and are independent of the impact of chronic pain or other sequelae. Dispositional traits contrast with the other key variables studied (e.g., anxiety, depression, and QoL) which are more transient emotional and outcome states. In previous work, we found that personality traits such as EA and AS were associated with increased disability and reduced coping [4]; the current study has now shown that they are also associated with higher doses of opioid analgesics.

Given that personality traits are relatively stable, the association with higher-dose opioids requires explanation. Given that previous work has shown that mood disorders and disability are higher in individuals with greater EA and AS, but not necessarily greater pain severity [4, 5], it is likely that the greater anxiety and depressive symptoms reported in our higher opioid dose group are related to the challenges in coping with pain-related disability. A commonly accepted explanation for the association between mood disorders and higher opioid doses is that higher doses of opioids are associated with poorly controlled pain and those with poorly controlled pain have high psychological distress. Our findings suggest that poorly controlled pain associated with higher doses of opioid analgesics is not due to greater pain severity but rather greater pain interference and lower QoL. Pain severity was not associated with higher doses of opioids in this study. The findings suggest that AS and EA are maladaptive with respect to coping with chronic pain limitations (i.e., pain interference) and not pain severity; as a result, these individuals are driven to seek out treatment solutions more aggressively, such as using higher doses of opioids, as a means of addressing the chronic pain limitations or pain interference.

Our findings with respect to the CAGE-AID further support the notion that the use of high-dose opioids is driven more by a need to try to deal with chronic pain limitations/interference and not a manifestation of problematic substance use. When answered honestly, the CAGE-AID is not a diagnostic tool; it is a highly sensitive and specific screen for problematic substance use. The current study found no difference in CAGE-AID scores between groups after controlling for average pain severity; further, over three-quarters of patients scored in the “no risk” group. It appears that high-dose opioid use was not associated with greater problematic substance use as identified by the CAGE-AID screen. However, it is worth noting that 8.5% of individuals in the high-dose opioid group had missing scores; this could potentially skew the data and is a limitation in fully evaluating this patient group.

Few studies have explored personality traits and mood with respect to opioid dosing. It is difficult to compare our findings to older studies, which were published in an era with fewer constraints on opioid prescribing practices [26]. Previous research has typically studied the general chronic pain population, irrespective of opioid use [14–17], or specifically among those who misuse opioids [18–22]. In the scientific literature, there has been a reliance on retrospective data collected from administrative datasets that have a narrow focus and are limited to demographic and clinical factors. As such, studies which evaluate patient-reported outcomes in association with opioid dose escalation are limited. Among recent studies, Morasco et al. [27] evaluated 517 individuals with chronic pain for differences in pain-related factors (i.e., severity, function), quality of life, and mental health based on three dose groups (i.e., 5.0–20.0 mg, 20.1–50.0 mg, and 50.1–120.0 mg). Morasco et al. [27] reported that the high-dose group had significantly greater pain severity, more impairments in functioning and quality of life, poorer self-efficacy for managing pain, greater fear avoidance, and more health care utilization. This study, unlike our study, found that high-dose opioid patients had greater pain severity but the remainder of the findings were compatible with our study.

Taking advantage of our earlier work examining the role of personality in chronic pain, this study was able to examine these same variables (EA and AS) as markers of individuals already identified at high risk of not coping well with chronic pain and, in particular, pain-related limitations, as identified by reports of pain interference. Our findings lend support toward treatment of chronic pain that targets maladaptive personality traits and dysfunctional coping such as cognitive behavioral therapy, pacing, and acceptance of physical limitations; these treatments have gained significant traction in the management of chronic pain but are poorly resourced in a public health care system more focused on medications, physical therapies, injections, and even invasive surgeries such as spinal cord stimulators. Treatment of psychological factors such as anxiety and depression (e.g., antidepressants and counseling) is also important. A large review has shown that strategies such as cognitive behavioral therapy are as effective in helping patients cope with pain as, ironically, interventions to target the pain itself (i.e., opioids) and they have the greater benefit of improving quality of life [28]. Cognitive-behavioral approaches may, therefore, potentially decrease and mitigate the need/demand for higher opioid dosing. As such, future studies should consider evaluating the comparative effects of cognitive-behavioral versus opioid analgesic interventions among those with chronic pain.

As mentioned above, nonpharmacological methods for addressing pain and coping include pacing activities, cognitive behavioral therapy [29], dialectical behavioral therapy [30], acceptance and commitment therapy [31], and mindfulness [32]. The current study provides credence to the importance of identifying and addressing mental health issues and maladaptive personality traits among chronic pain patients taking opioids, particularly those utilizing higher doses, and providing coping supports that target high-risk groups. Finally, the findings may give support and direction to public policy for the treatment of chronic pain, particularly those on opioid analgesics as part of a gradual reduction strategy.

The findings from this study have generated additional questions that are valuable to investigate in the future. The last decade has seen a definitive trend away from high-dose opioids in the treatment of chronic pain, and over the last five years, there have been sustained efforts to reduce the dosage of opioids. This has been reinforced by clinical guidelines which set maximum recommended doses. Unfortunately, physical dependency, anxiety over potential withdrawal, and reluctance to reduce a stable dose of opioids have made high-dose opioid withdrawal very difficult [33]. This is particularly true for those who are at high risk and are coping poorly with pain-related limitations or concurrent diagnosis of opioid use disorder. In the case of the latter, medication-assisted treatment may be necessary for the successful tapering of high-dose opioids. Important next steps may include prospective interventional studies which aim to address maladaptive personality traits, particularly those which impact pain interference by decreasing psychological distress.

### 4.1. Limitations

This study is limited as data was collected cross-sectionally, at a single site with small sample size. It is possible that the individuals recruited to this study represent a subset of the chronic pain population, specifically, those requiring referral to specialist care. This cohort may have more significant issues that require ongoing assessment and support, in contrast to individuals with chronic pain that do not require specialist referral. Future studies may overcome sample size limitations by recruiting individuals from primary care practices who are experiencing a primary complaint of pain. Two individuals who were approached to participate in this study declined; unfortunately, personal information and reason for declination were not available for these individuals, so it is not possible to characterize them. At the time of data collection, certain personal and health information was not collected which would have provided greater context (e.g., socioeconomic status, history of psychological/physical abuse, opioid naiveté, side effects profiles, nonopioid use, medical comorbidities, use of social assistance, litigation status, and social support); these factors, not considered for the study, may have an important role in mediating the use of opioids in those with chronic pain.

## 5. Conclusion

EA and AS are two personality traits that may contribute to psychological distress and the use of higher-dose opioids given their role in maladaptive coping to chronic pain. Our study found that after controlling for average pain severity, patients with MEQ 90+ mg had significantly greater scores of EA and AS, as well as higher scores on pain interference, with poorer mood, coping, and QoL than those with MEQ 1–89 mg or MEQ 0 mg; there was no association with risk of substance misuse. Maladaptive personality traits are correlated with a higher risk of psychological distress, poor coping, and greater pain interference which in turn is associated with the use of higher-dose opioids; these all need to be addressed as part of a chronic pain treatment plan.

## Figures and Tables

**Table 1:** Sociodemographic information and descriptive statistics for the total sample and three morphine equivalent groups.

Variable	Total	MEQ 0 mg	MEQ 1–89 mg	MEQ 90+ mg	*p*
	*N* = 215	*N* = 58	*N* = 86	*N* = 71	
Mean age ±SD (range) years	52.7 ± 11.7	52.1 ± 14.7	52.7 ± 11.4	53.2 ± 9.3	0.868

Gender					0.001
Males	77 (35.8%)	12 (20.7%)	28 (32.6%)	37 (52.1%)	
Females	138 (64.2%)	46 (79.3%)	58 (67.4%)	34 (47.9%)	

Employment					0.001
Full-time	35 (16.3%)	13 (22.4%)	18 (20.9%)	4 (5.6%)	
Part-time	12 (5.6%)	6 (10.3%)	6 (7.0%)	0 (0.0%)	
Casual	9 (4.2%)	4 (6.9%)	3 (3.5%)	2 (2.8%)	
Temporary	1 (0.5%)	0 (0.0%)	0 (0.0%)	1 (1.4%)	
Unemployed/retired	137 (63.7%)	35 (60.3%)	46 (53.5%)	56 (78.9%)	
Unknown	21 (9.8%)	0 (0.0%)	13 (15.1%)	8 (11.3%)	

Education					0.424
Did not graduate from high school	32 (14.9%)	8 (13.8%)	10 (11.6%)	14 (19.7%)	
Highschool graduate	45 (20.9%)	14 (24.1%)	13 (15.1%)	18 (25.4%)	
Some postsecondary education	67 (31.2%)	17 (29.3%)	31 (36.0%)	19 (26.8%)	
Bachelor's degree	42 (19.5%)	15 (25.9%)	17 (19.8%)	10 (14.1%)	
Graduate school	9 (4.2%)	4 (6.9%)	3 (3.5%)	2 (2.8%)	
Unknown	20 (9.3%)	0 (0.0%)	12 (14.0%)	8 (11.3%)	

Living arrangement					0.558
House	133 (61.9%)	43 (74.1%)	49 (57.0%)	41 (57.7%)	
Apartment/condominium	60 (27.9%)	15 (25.9%)	25 (29.1%)	20 (28.2%)	
Retirement/assisted living	0 (0.0%)	0 (0.0%)	0 (0.0%)	0 (0.0%)	
Homeless	1 (0.5%)	0 (0.0%)	0 (0.0%)	1 (1.4%)	
Unknown	21 (9.8%)	0 (0.0%)	12 (14.0%)	9 (12.7%)	

Race					0.276
Caucasian	171 (79.5%)	50 (86.2%)	68 (79.1%)	53 (74.6%)	
Other	44 (20.5%)	8 (13.8%)	18 (20.9%)	18 (25.4%)	

MEQ = morphine equivalent.

**Table 2:** Clinical health information and descriptive statistics for the total sample and three morphine equivalent groups.

Variable	Total	MEQ 0 mg	MEQ 1–89 mg	MEQ 90+ mg	*p*
	*N* = 215	*N* = 58	*N* = 86	*N* = 71	
Time since injury (years)	14.1 ± 10.2	8.6 ± 81	14.0 ± 10.1	18.9 ± 9.8	<0.001^a,b,c^

Length of clinic enrolment (years)	7.4 ± 7.7	3.1 ± 4.7	7.5 ± 7.9	11.3 ± 7.7	<0.001^a,b,c^

Type of pain					0.398
Nociceptive	78 (36.3%)	22 (37.9%)	33 (38.4%)	23 (32.4%)	
Neuropathic	28 (13.0%)	4 (6.9%)	15 (17.4%)	9 (12.7%)	
Mixed	102 (47.4%)	30 (51.7%)	34 (39.5%)	38 (53.5%)	
Unknown	7 (3.3%)	2 (3.4%)	4 (4.7%)	1 (1.4%)	

Opioid agents used^∗^					
Hydromorphone/contin	32 (14.9%)	N/A	14 (16.3%)	18 (25.4%)	0.170
Oxycontin/Oxyneo	39 (18.1%)		8 (9.3%)	31 (43.7%)	<0.001
Tramadol/Tramacet	19 (8.8%)		18 (20.9%)	1 (1.4%)	<0.001
Percocet	68 (31.6%)		32 (37.2%)	36 (50.7%)	0.106
Morphine/contin	38 (17.7%)		15 (17.4%)	23 (32.4%)	0.039
Fentanyl	12 (5.6%)		2 (2.3%)	10 (14.1%)	0.007
Tylenol 3 or 4	15 (7.0%)		14 (16.3%)	1 (1.4%)	0.002
Methadone	2 (0.9%)		0 (0.0%)	2 (2.8%)	0.203
Butrans	1 (0.5%)		1 (1.2%)	0 (0.0%)	1.00

Use of nonopioid medications					0.862
Yes	64 (29.8)	19 (32.8%)	25 (29.1%)	20 (28.2%)	
No	151 (70.2)	39 (67.2%)	61 (70.9%)	51 (71.8%)	

Morphine equivalent dose					N/A
Mean ± standard deviation	85.0 ± 123.2 (0–745)	N/A	36.0 ± 24.2 (2–88)	212.4 ± 142.9 (90–745)	
Median (interquartile range)	40.0 (0–114)		35.5 (14.6–60.0)	165.0 (114–255)	

Cannabis prescribed					0.754
Yes	63 (29.3%)	17 (29.3%)	23 (26.7%)	23 (32.4%)	
No	149 (69.3%)	41 (70.7)	61 (70.9%)	47 (66.2%)	
Unknown	3 (1.4%)	0 (0.0%)	2 (2.3%)	1 (1.4%)	

^a^MEQ = 0 mg vs. MEQ 1–89 mg significantly different (*p* < .05); ^b^MEQ = 0 mg vs. MEQ 90+ mg significantly different (*p* < .05); ^c^MEQ = 1–89 mg vs. MEQ 90+ mg significantly different (*p* < .05); ^∗^Sum >215 as some patients were taking >1 opioid; MEQ = morphine equivalent.

**Table 3:** Outcome measures.

Variable	Total	MEQ 0 mg	MEQ 1–89 mg	MEQ 90+ mg	ANCOVA
	*N* = 215	*N* = 58	*N* = 86	*N* = 71	*p*
Brief Pain Inventory Short Form					
Average pain (item 5) mean score	6.2 ± 2.0	5.5 ± 2.0	6.1 ± 2.0	6.9 ± 1.8	<0.001^b,c^
Pain severity (items 3–6) mean score	24.7 ± 7.5	22.5 ± 7.4	24.0 ± 8.0	27.5 ± 5.8	0.337
Pain interference (items 9a-g) mean score	44.3 ± 17.3	37.1 ± 18.8	41.9 ± 16.5	53.1 ± 13.0	<0.001^b,c^

AAQ mean score	34.7 ± 9.7	32.5 ± 8.7	34.9 ± 9.8	36.8 ± 10.2	0.008^b^

ASI mean score	24.1 ± 13.3	21.3 ± 12.6	22.1 ± 12.8	29.3 ± 13.2	0.001^b,c^
ASI = 0–7 (none)	13 (6.0%)	4 (6.9%)	6 (7.0%)	3 (4.2%)	
ASI = 8–15 (mild)	46 (21.4%)	20 (34.5%)	19 (22.1%)	7 (9.9%)	
ASI = 16–25 (moderate)	55 (25.6%)	15 (25.9%)	24 (27.9%)	16 (22.5%)	
ASI = 26–63 (severe)	78 (36.3%)	19 (32.8%)	25 (29.1%)	34 (47.9%)	
Missing	23 (10.7%)	0 (0.0%)	12 (14.0%)	11 (15.5%)	

PHQ-9 mean score	13.4 ± 6.9	10.4 ± 6.4	12.6 ± 6.8	17.0 ± 5.8	<0.001^b,c^
PHQ-9 = 0–4 (none-minimal)	27 (12.6%)	13 (22.4%)	12 (14.0%)	2 (2.8%)	
PHQ-9 = 5–9 (mild)	44 (20.5%)	15 (25.9%)	20 (23.3%)	9 (12.7%)	
PHQ-9 = 10–14 (moderate)	38 (17.7%)	14 (24.1%)	18 (20.9%)	6 (8.5%)	
PHQ-9 = 15–19 (moderately severe)	60 (27.9%)	11 (19.0%)	20 (23.3%)	29 (40.8%)	
PHQ-9 = 20–27 (severe)	43 (20.0%)	5 (8.6%)	15 (17.4%)	23 (32.4%)	
Missing	3 (1.4%)	0 (0.0%)	1 (1.2%)	2 (2.8%)	

GAD-7 mean score	9.9 ± 6.0	8.4 ± 5.5	9.2 ± 5.8	12.2 ± 6.0	0.009^b,c^
GAD-7 = 0–5 (none)	57 (26.5%)	18 (31.0%)	27 (31.4%)	12 (16.9%)	
GAD-7 = 6–10 (mild)	56 (26.0%)	18 (31.0%)	24 (27.9%)	14 (19.7%)	
GAD-7 = 11–15 (moderate)	56 (26.0%)	14 (24.1%)	20 (23.3%)	22 (31.0%)	
GAD-7 = 16–21 (severe)	43 (2.0%)	8 (13.8%)	14 (16.3%)	21 (29.6%)	
Missing	3 (1.4%)	0 (0.0%)	1 (1.2%)	2 (2.8%)	

CAGE-AID mean score	0.6 ± 1.0	0.2 ± 0.4	0.5 ± 1.0	0.6 ± 1.0	0.868
CAGE-AID = 0–1 (no risk)	169 (78.6%)	45 (77.6%)	69 (80.2%)	55 (77.5%)	
CAGE-AID = 2–4 (risk)	34 (15.8%)	11 (19.0%)	13 (15.1%)	10 (14.1%)	
Missing	12 (5.6%)	2 (3.4%)	4 (4.7%)	6 (8.5%)	

EQ5D VAS mean score	51.3 ± 20.0	57.9 ± 20.1	53.3 ± 20.0	43.4 ± 17.4	0.004^b,c^

Brief COPE-28					
Problem-based coping mean score	16.5 ± 4.4	16.6 ± 4.1	16.8 ± 4.4	16.1 ± 4.7	0.706
Emotion-focused coping mean score	25.0 ± 6.0	24.9 ± 6.2	24.9 ± 5.9	25.2 ± 5.9	0.997
Dysfunctional coping mean score	21.2 ± 5.5	20.4 ± 4.4	20.6 ± 6.0	22.6 ± 5.7	0.008^b^

AAQ = Acceptance and Action Questionnaire; ASI = Anxiety Sensitivity Index; Brief COPE-28 = Brief- Coping with Problems Experience 28 item; BPI-SF = Brief Pain Inventory Short Form; CAGE-AID = substance abuse screening tool; EQ5D VAS = EuroQol-5D Visual Analogue Scale; GAD-7 = Generalized Anxiety Disorder 7 item; PHQ-9 = Patient Health Questionnaire 9 item (PHQ-9).

## Data Availability

The data are available from the corresponding author upon request.

## References

[1] Schopflocher D., Taenzer P., Jovey R. (2011). The prevalence of chronic pain in Canada. *Pain Research and Management*.

[2] Merskey H. (1994). *Classification of Chronic Pain*.

[3] Rodríguez I., Abarca E., Herskovic V., Campos M. (2019). Living with chronic pain: a qualitative study of the daily life of older people with chronic pain in Chile. *Pain Research & Management*.

[4] Mehta S., Rice D., Janzen S. (2016). The long term role of anxiety sensitivity and experiential avoidance on pain intensity, mood, and disability among individuals in a specialist pain clinic. *Pain Research & Management*.

[5] Mehta S., Rice D, McIntyre A (2016). Identification and characterization of unique subgroups of chronic pain individuals with dispositional personality traits. *Pain Research & Management*.

[6] Shields G. S., Toussaint L. L., Slavich G. M. (2016). Stress-related changes in personality: a longitudinal study of perceived stress and trait pessimism. *Journal of Research in Personality*.

[7] Bleidorn W. (2012). Hitting the road to adulthood. *Personality and Social Psychology Bulletin*.

[8] Hildebrandt J. (2014). Prediction of psychosocial factors by pain drawing in patients with chronic back pain. *Pain Medicine*.

[9] Furlan A. D. (2006). Opioids for chronic noncancer pain: a meta-analysis of effectiveness and side effects. *Canadian Medical Association Journal*.

[10] Busse J. W., Craigie S., Juurlink D. N. (2017). Guideline for opioid therapy and chronic noncancer pain. *Canadian Medical Association Journal*.

[11] Vowles K. E., McEntee M. L., Julnes P. S., Frohe T., Ney J. P., van der Goes D. N. (2015). Rates of opioid misuse, abuse, and addiction in chronic pain. *Pain*.

[12] Chou R., Clark E., Helfand M. (2003). Comparative efficacy and safety of long-acting oral opioids for chronic non-cancer pain: a systematic review. *Journal of Pain and Symptom Management*.

[13] Eisenberg E., McNicol E. D., Carr D. B. (2005). Efficacy and safety of opioid agonists in the treatment of neuropathic pain of nonmalignant origin. *JAMA*.

[14] Poppe C. (2011). Personality traits in chronic pain patients are associated with low acceptance and catastrophizing about pain. *Acta Clinica Belgica*.

[15] Knaster P., Estlander A.-M., Karlsson H., Kaprio J., Kalso E. (2012). Temperament traits and chronic pain: the association of harm avoidance and pain-related anxiety. *PLoS One*.

[16] Koh J. S., Ko H. J., Wang S.-M. (2014). The association of personality trait on treatment outcomes in patients with chronic prostatitis/chronic pelvic pain syndrome: an exploratory study. *Journal of Psychosomatic Research*.

[17] Kadimpati S., Zale E. L., Hooten M. W., Ditre J. W., Warner D. O. (2015). Associations between neuroticism and depression in relation to catastrophizing and pain-related anxiety in chronic pain patients. *PLoS One*.

[18] Rogers A. H., Bakhshaie J., Zvolensky M. J., Vowles K. E. (2020). Pain anxiety as a mechanism linking pain severity and opioid misuse and disability among individuals with chronic pain. *Journal of Addiction Medicine*.

[19] Rogers A. H., Shepherd J. M., Orr M. F., Bakhshaie J., McHugh R. K., Zvolensky M. J. (2019). Exploring anxiety sensitivity in the relationship between pain intensity and opioid misuse among opioid-using adults with chronic pain. *Journal of Psychiatric Research*.

[20] Zvolensky M. J., Rogers A. H., Shepherd J. M., Vujanovic A. A., Bakhshaie J. (2020). Anxiety sensitivity and opioid misuse and dependence among trauma-exposed adults with chronic pain. *Journal of Behavioral Medicine*.

[21] Feingold D., Brill S., Goor-Aryeh I., Delayahu Y., Lev-Ran S. (2018). The association between severity of depression and prescription opioid misuse among chronic pain patients with and without anxiety: a cross-sectional study. *Journal of Affective Disorders*.

[22] Feingold D., Brill S., Goor-Aryeh I., Delayahu Y., Lev-Ran S. (2017). Misuse of prescription opioids among chronic pain patients suffering from anxiety: a cross-sectional analysis. *General Hospital Psychiatry*.

[23] Eriksen J., Sjøgren P., Bruera E., Ekholm O., Rasmussen N. K. (2006). Critical issues on opioids in chronic non-cancer pain: an epidemiological study. *Pain*.

[24] Keane M. (2007). Caution with epidemiological data in relation to chronic opioid use. *Pain*.

[25] Damian R. I., Spengler M., Sutu A., Roberts B. W. (2019). Sixteen going on sixty-six: a longitudinal study of personality stability and change across 50 years. *Journal of Personality and Social Psychology*.

[26] Kobus A. M., Smith D. H., Morasco B. J. (2012). Correlates of higher-dose opioid medication use for low back pain in primary care. *The Journal of Pain*.

[27] Morasco B. J., Yarborough B. J., Smith N. X. (2017). Higher prescription opioid dose is associated with worse patient-reported pain outcomes and more health care utilization. *The Journal of Pain*.

[28] Majeed M. H., Sudak D. M. (2017). Cognitive behavioral therapy for chronic pain-one therapeutic approach for the opioid epidemic. *Journal of Psychiatric Practice*.

[29] Niknejad B., Bolier R., Henderson C. R. (2018). Association between psychological interventions and chronic pain outcomes in older adults. *JAMA Internal Medicine*.

[30] Linton S. J. (2010). Applying dialectical behavior therapy to chronic pain: a case study. *Scandinavian Journal of Pain*.

[31] Hughes L. S., Clark J., Colclough J. A., Dale E., McMillan D. (2017). Acceptance and commitment therapy (ACT) for chronic pain. *The Clinical Journal of Pain*.

[32] Hilton L., Hempel S., Ewing B. A. (2017). Mindfulness meditation for chronic pain: systematic review and meta-analysis. *Annals of Behavioral Medicine*.

[33] Goesling J., DeJonckheere M., Pierce J. (2019). Opioid cessation and chronic pain: perspectives of former opioid users. *Pain*.

